# The energy dependence of contrast and damage in electron cryomicroscopy of biological molecules

**DOI:** 10.1016/j.ultramic.2019.02.007

**Published:** 2019-08

**Authors:** Mathew J. Peet, Richard Henderson, Christopher J. Russo

**Affiliations:** MRC Laboratory of Molecular Biology, Francis Crick Avenue, Cambridge CB2 0QH, UK

**Keywords:** Radiation damage, Electron scattering cross-section, Low-dose electron microscopy, cryoEM, Structure determination

## Abstract

We have measured the dependence on electron energy of elastic and inelastic scattering cross-sections from carbon, over the energy range that includes 100 keV to 300 keV. We also compared quantitatively the radiation damage to bacteriorhodopsin and paraffin (C_44_H_90_) at 100 keV and 300 keV by observing the fading of the diffraction spots from two-dimensional crystals as a function of electron fluence. The elastic cross-section is 2.01 - fold greater at 100 keV than at 300 keV, whereas the radiation damage increased by only 1.57. This implies that the amount of useful information from diffraction patterns or images of most biological structures should be 25% greater using 100 keV rather than 300 keV electrons. Using these measurements, we calculate the energy dependence of the available information per unit damage for a specimen of a particular thickness, which we call the “information coefficient.” This allows us to determine the optimal energy for imaging a biological specimen of a given thickness. We find that for most single particle cryoEM specimens, 100 keV provides not only the highest potential for information per unit damage, but would also simplify the instrument while retaining the potential to reach high resolution with a minimum of data. These measurements will help guide the development and use of electron cryomicroscopes for biology.

## Highlights

• Carbon elastic and inelastic electron scattering cross sections are measured vs. energy.
• Elastic scattering is compared to energy deposition and radiation damage.
• An optimal energy for cryoEM of a given biological specimen thickness is determined.

## 1 Introduction

Specimen damage is the fundamental limit to all forms of microscopy capable of atomic resolution imaging. Particularly for electron microscopy of biological specimens – comprised of RNA, DNA, proteins and other organic macromolecules in an aqueous environment – cumulative ionisation damage and bond breakage limits the available information that can be obtained by any imaging technique before the specimen is destroyed. Low temperature techniques reduce the damaging effects of ionising radiation and are effective in both electron microscopy and X-ray crystallography [1], [2]. Although radiation damage is what limits the usable electron fluence at all specimen temperatures, recent improvements in both software and hardware technology for electron cryomicroscopy (cryoEM), including fast and efficient detectors, new algorithms for image processing, and methods to reduce the movement of the specimen during imaging have all contributed to increases in the signal-to-noise ratio in micrographs collected [3]. It is with this in mind that we revisit the fundamental question of the amount of information available per unit damage caused by irradiation with high energy electrons. We then discuss these measurements in the context of current and future technology for electron cryomicroscopy of biological specimens.

Since the energy of the incident electron is precisely controllable over the range of interest to transmission electron microscopy, 1 keV to 10 MeV, it is of interest to understand how the choice of accelerating voltage affects image quality in cryoEM. While the optics of the microscope may limit the choice of energy for a variety of reasons, the last two decades of development in electron microscope hardware have meant that in principle and in practice, a microscope capable of sub-two Ångstrom resolution can be constructed at energies ranging from 20 keV to 3 MeV [4], [5]. The natural question is then: *what is the best energy for cryoEM?* Put in another way, if the non-trivial issues of hardware are set aside and consideration is given only to beam–specimen interaction, what electron energy might provide the best images? To answer these questions, we have determined how the amount of damage to the specimen changes with the energy of the incident electron beam, and in turn how this compares to the scattering contrast available for high resolution imaging.

Previous calculations of the relationship between the elastic and inelastic scattering estimated that the two quantities parallel each other to a reasonable approximation [6], [7], [8], [9], [10]. Technology has progressed and now the success of cryoEM methods obliges us to reconsider this relationship with more precision. More complete calculations of the inelastic interactions of electrons with atoms in the specimen indicate that there should be a difference in the energy dependence in comparison to elastic scattering, in that the inelastic cross-section scales more slowly than the inverse of the energy of the electron [11], [12]. Still, the available measurements of these cross-sections to date are of insufficient accuracy to verify these theoretical differences in the range of energies of interest to cryoEM ( ∼ 60–300 keV) [13], [14], [15], [16]. Others have recently reported successfully determining high resolution structures with 200 keV electrons, but without a quantitative evaluation of the various factors involved like electron source coherence, detector efficiency and relative amounts of radiation damage [17]. Furthermore, a definitive relationship between the inelastic scattering cross-section for carbon and the amount of damage to a protein specimen has not been measured. In this work we endeavour to accurately measure the elastic and inelastic cross-sections as a function of energy, and compare these to a quantitative measurement of the rate of damage to biological specimens at cryogenic temperatures. These measurements then allow us to verify the theoretical relationship between electron beam energy and information per unit damage to biological specimens; thus we are able to determine the appropriate energy for most single particle cryoEM investigations.

## 2 Materials and methods

### 2.1 Elastic and inelastic scattering from carbon

#### 2.1.1 Specimen preparation

Single layer graphene produced by chemical vapour deposition was suspended over the holes in a gold specimen support (gold foil on gold grid) Quantifoil UltrAuFoil R 1.2/1.3 [18], [19]. Amorphous carbon was evaporated onto a mica sheet and transferred to 300 lines/inch hexagonal copper mesh grids by flotation on water. Freshly cleaved mica was positioned 125 mm from a carbon source in Edwards Turbo 306 evaporator. After pumping to $2\times 10^{-7}$ mbar, deposition of carbon from the source was controlled using a crystal thickness monitor (Inficon QM-160). By cleaving an area of amorphous carbon from the mica, the average thickness was measured to be 45.89  ±  0.03 Å using an Asylum Research atomic force microscope in direct contact mode. We note that in practice the accuracy of the measurement of the thickness of the carbon film was limited not by this measurement but instead by contamination or etching in the electron microscope, which was about 5% as estimated by the measured contamination rate in the column. The density of the amorphous carbon film, 2.21  ±  0.11 g/cm^3^, was determined by adding flakes of the film to a columnar density gradient of bromoform and chloroform and allowing the flakes to settle for several hours.

#### 2.1.2 Data collection

**Fig. 1:**
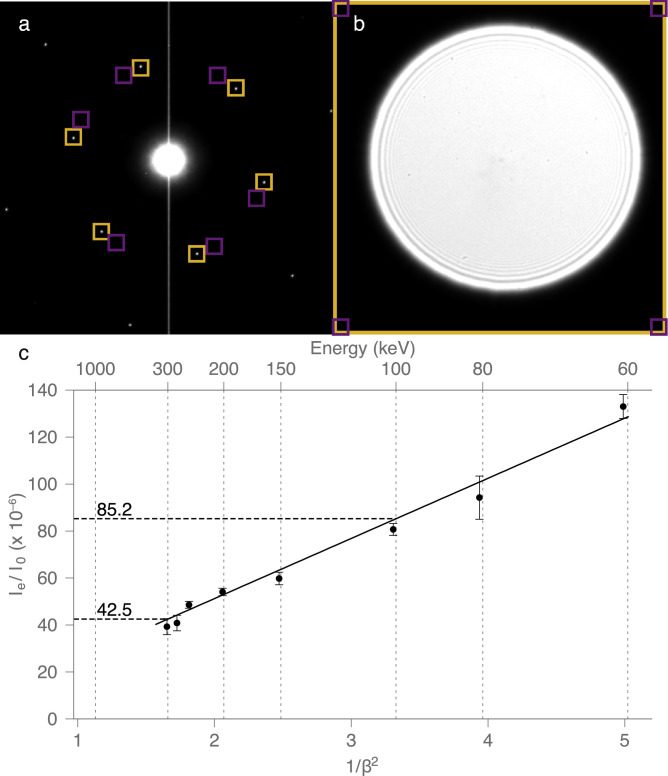
Elastic scattering cross-section vs. energy for carbon in the form of graphene. A typical diffraction pattern is shown in (a) where the orange boxes surround the first order Bragg reflections at 1/2.14 Å${}^{-1}$. To measure the intensity of this peak (taken as *I_e_*, the intensity of elastic scattering), the sum of the pixel values in the box was subtracted from an adjacent box (purple) at the same radius. This was then compared to the forward scattered central beam intensity *I*_(0,0)_ using *I*_0_ = *I_e_* + *I*_(0,0)_ (b) defocusing the (0,0) spot prevented saturation of the detector and background intensity was determined from measurements at the corners of the image (of the diffraction pattern). The ratio of the first reflection to the forward scattered beam is plotted vs 1/*β*^2^ in (c), where *β* is the ratio of the electron speed to that of light and the error bars are the standard deviation of three measurements at each energy.

Elastic scattering intensity was measured using diffraction from single layer graphene using an FEI Polara G2 transmission electron microscope with specimens cooled to  ∼ 80K at accelerating voltages between 60 keV and 300 keV at camera lengths between 250 mm and 470 mm on a Gatan Orius SC1000 CCD with an area of 2048  ×  2048 pixel. The intensity of diffraction was taken as the average value of 5 or 6 first order diffraction spots of graphene, measured using 64  ×  64 pixel boxes, subtracting the background for each from an adjacent area of the same dimensions, radial distance, and detector quadrant. Defocusing the diffraction pattern a second image was recorded to measure the transmitted intensity (0,0) spot whilst avoiding saturation of the detector pixels. Boxes of 64  ×  64 pixel in each corner were taken as the average background which was subtracted from the integrated intensity of the whole image of the defocused transmitted beam. Fig. 1 demonstrates the measurement scheme for (a) the first order diffraction spots and (b) the defocused (0,0) spot.

**Fig. 2:**
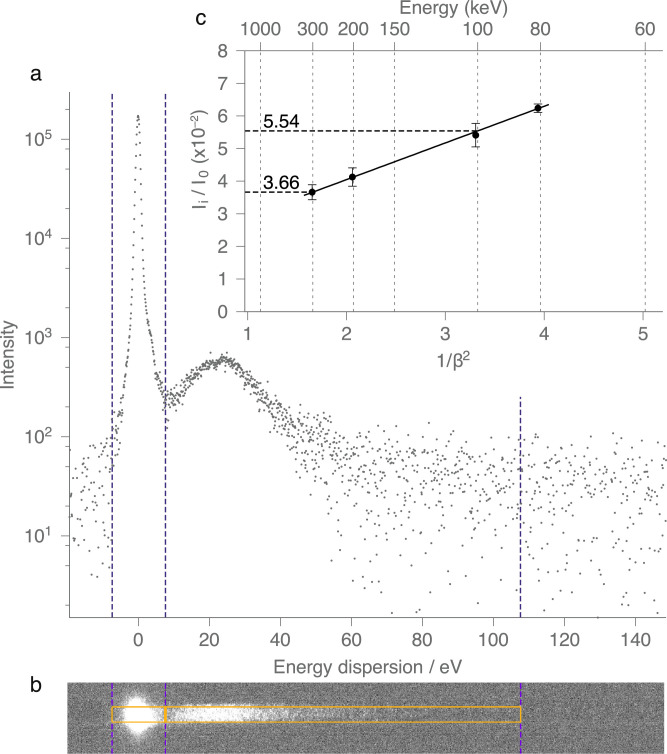
Inelastic scattering cross-section vs. energy for amorphous carbon. Panels (a) & (b) example spectra taken at 80 keV illustrates how the inelastic scattering intensity at each operating voltage was determined by integration of the zero loss peak and the inelastic scattering in a window that is 100 eV wide. Plot (c) shows the ratio of the inelastic scattering intensity to the zero loss peak at 4 different energies, plotted vs 1/*β*^2^.

Inelastic scattering from a thin film of amorphous carbon was measured using a Gatan EELS 932C (864 GIF Tridium) spectrometer with US4000 CCD. We determined the intensity ratio of the zero loss peak to the energy loss peak as shown in Fig. 2(a) and (b) at several acceleration voltages (c). As shown in (a) the intensity asymptotically approaches the background. Robust measurement was achieved by determining the position of minima between the two peaks and integrating the intensity in a 100 eV energy window for the energy loss peak. Background subtraction was achieved by measuring intensity from areas of the same size above the spectrum on the CCD for the zero loss peak and the energy loss peak. At each accelerating energy this procedure was repeated at least three times at different locations to assess the measurement uncertainty. The energy dispersion was calibrated using the plasmon peak position collected from a thin foil of aluminium (EMS Catalogue #80044).

Simultaneous data collection for both the zero loss peak and the energy loss peak was applied to eliminate any effects of varying beam current. This was achieved by integrating a series of spectra acquired by reading out the full CCD and limiting the intensity of the zero loss peak in each image to the linearised response range of intensity. The maximum intensity per image for the data included in our analysis varied from 2000 to 14,000 counts, whereas no measurable difference in intensity ratio between energy loss peak and zero loss peak could be detected with a maximum intensity of 22,000 counts per frame. For each experiment the exposure per frame varied from 0.5-1 s, cropped images including the spectra and area for background measurement were aligned and integrated, giving a total acquisition time from 60 to 150 s. Electron fluence ranged from 0.4 to 10 e${}^{-}$/nm^2^, with a beam of area 40–440 µm^2^. The selected area aperture and entrance aperture limited the collection area and semi-angle, which varied from 1 to 2 mm at the entrance aperture and from 9 to 27 mrad.

### 2.2 Radiation damage to 2D crystals

#### 2.2.1 Specimen preparation

Specimens of C_44_H_90_ paraffin were made by applying 2 µl of a nearly saturated solution of C_44_H_90_ from Supelco Inc. in hexane to a film of amorphous carbon that had previously been floated off mica on to 400 lines/inch copper EM grids, and allowed to dry in air. Crystals of C_44_H_90_ paraffin one molecule thick formed as described in detail in [20]. Specimens of purple membrane (bacteriorhodopsin 2D crystals) were prepared as described in [21]. Briefly, 400 lines/inch grids were coated with amorphous carbon, that had been floated off mica. These grids were pre-treated with a 4 µl drop of 1% ovalbumin (Sigma), then washed with 2 drops of water then dried. A 2 µl drop of fused purple membranes (1 mg/ml) was then applied to the pre-treated grids, blotted and then, while still wet, a final 2 µl drop of 1% glucose was applied, blotted and dried in air.

#### 2.2.2 Electron diffraction

Electron diffraction patterns were recorded at 100 keV or at 300 keV on an FEI Polara G2, with the specimens cooled to liquid nitrogen temperature, using a camera length of 930 mm. The beam current, calibrated at both 100 keV and 300 keV using a Faraday cup and picoammeter, was between 90 and 120 pA and the illuminated area approximately 1 µm in diameter (measured accurately using a cross grating replica for callibration of magnification). For C_44_H_90_ paraffin at 100 keV, a series of 12  ×  0.4 s exposures was acquired at a flux of 1.70 $e^{-}/$Å^2^/frame, with the diffraction patterns slightly defocussed to avoid saturating the detector, a Gatan Orius SC200B. For C_44_H_90_ at 300 keV, a series of 150  ×  0.1 s exposures was acquired at a flux of 0.234 $e^{-}/$Å^2^/frame. For purple membrane at 100 keV, a series of 8  ×  0.8 s exposures at a flux of 3.42 $e^{-}/$Å^2^/frame was acquired. For purple membrane at 300 keV, a series of 8  ×  1.5 s exposures at a flux of 3.51 $e^{-}/$Å^2^/frame was acquired. The linearity of the exposure times was also checked and found to be accurate to 0.01%.

Electron diffraction patterns of purple membrane were processed as described previously [21], then the average intensities in three resolution zones, centred at 9 Å, 5 Å and 4 Å were plotted as a function of electron fluence as a ratio to the average intensity in the first frame. For C_44_H_90_ paraffin, the centres of the 5 or 6 individual spots with indices (1,1), (1,−1) and (2,0) and their corresponding Friedel mates were used to integrate a circle of background-corrected diffracted intensity for each spot on each frame as the diffraction spots faded.

## 3 Results

### 3.1 Carbon elastic scattering cross-section vs energy

Measurement of the energy dependence of the elastic scattering cross-section for the first ring of reflections in graphene is shown in Fig. 1. The upper panels (a and b) show representative diffraction patterns used to measure the ratio of the elastically scattered beam (*I_e_*) to the forward scattered (unscattered) beam (*I*_0_). The same reflections (5 or 6 measurements at 1/2.14 Å${}^{-1}$) were used at each energy and the average value taken as *I_e_*. Multiple diffraction patterns were collected at each energy, and were then plotted against 1/*β*^2^ where β=v/c, the ratio of the electron speed (*v*) to that of light (*c*). Values of the electron energy are also provided on the upper axis for convenience. The measurements show that the scattering into the first reflection scales linearly with 1/*β*^2^, and the ratio of the elastic scattering at 100 keV to 300 keV is 2.01  ±  0.05.

### 3.2 Carbon inelastic scattering cross-section vs energy

To measure the inelastic scattering, we use electron energy loss spectrometry, with a dispersion setting appropriate to capture the low loss spectra at different energies. In practice, this measurement was more difficult than the elastic measurement since it was very sensitive to the thickness of the specimen, including any form of systematic contamination that may be present on the test specimen. Initially we attempted to use the low-loss spectra of graphene for this measurement, but the plasmon losses in graphene are very sensitive to even small amounts of material adsorbed to the surface (the plasmon peak can shift by several volts with one monolayer attached [22]). Therefore, despite the interesting physics involved in this process, this proved to be too inaccurate for the purposes of this measurement. Instead, we used a thin film of amorphous carbon (46 Å) which was prepared to be thick enough to give sufficient signal in the low-loss regime but thin enough to render multiple inelastic scattering undetectable in the spectra. We also measured the density of the amorphous carbon (2.21  ±  0.11 g/cm^3^) to verify it was in the range expected for arc-evaporated films of this type and to allow comparison with absolute cross-sections previously reported. Spectra were collected at a range of energies from 80 to 300 keV, and the results are shown in Fig. 2. The spectra (Fig. 2a) were created from the images on the detector (Fig. 2b) by summing the columns at each energy channel and subtracting the background. The inelastic current and elastic currents, *I_i_* and *I*_0_ were then taken as the integral over the energy windows as indicated, and the ratio is plotted against 1/*β*^2^ in Fig. 2c. The relationship is again approximately linear with 1/*β*^2^, but the slope and intercept differ from those for elastic scattering. In particular, the ratio of inelastic scattering at 100 keV to 300 keV is 1.51  ±  0.1.

### 3.3 Radiation damage to 2D crystals

**Fig. 3:**
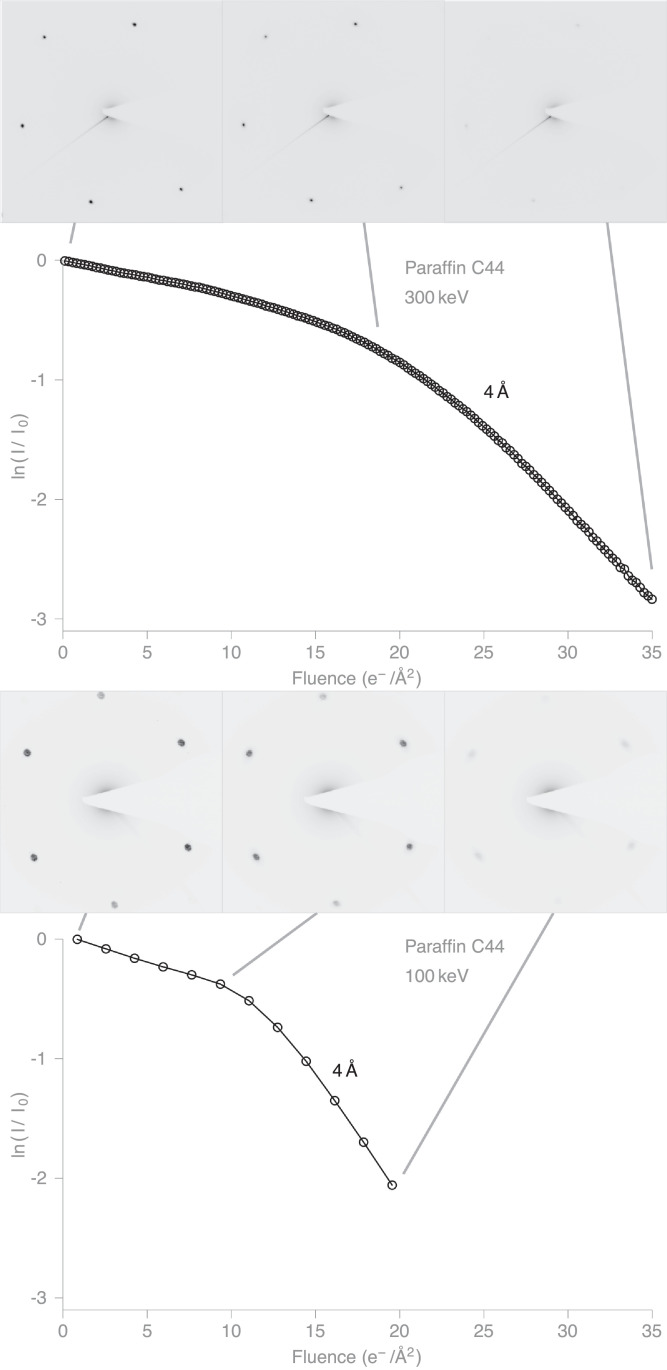
Diffraction series at 300 and 100 keV of paraffin (C_44_H_90_) 2D crystals at 80K. Plots show the average intensity of the  ∼ 1/4 Å${}^{-1}$ reflections vs. fluence at 300 and 100 keV. With spot intensities measured by integration. See also Supplementary Movies 1 & 2.

**Fig. 4:**
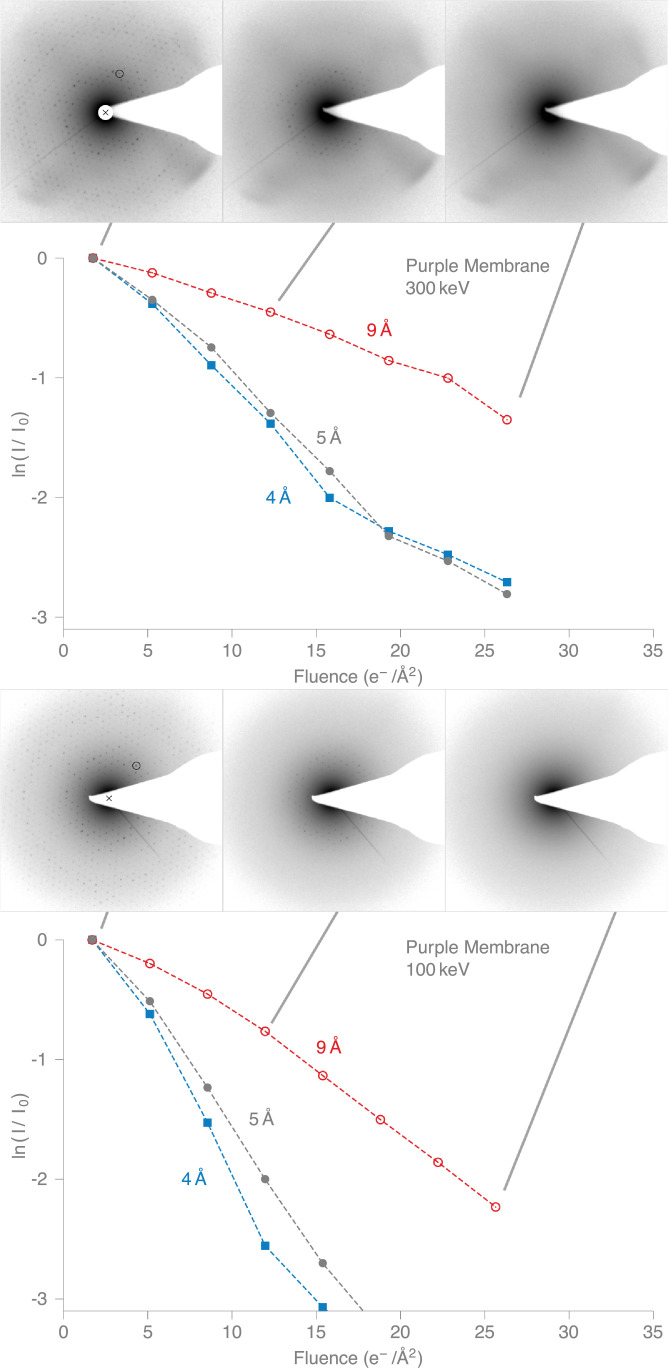
Diffraction series at 300 and 100 keV of purple membrane (bacteriorhodopsin) 2D crystals at 80K. Diffraction patterns show logarithm of intensity to enhance visibility. The first image in each sequence has the (4,3) purple membrane reflection circled and (0,0) location marked by cross. Plot shows the quantification of the total intensity in all the spots in a particular resolution range as indicated and described in the text. See also Supplementary Movies 3 & 4.

Fig. 3 shows the fading of the intensity of the C_44_H_90_ paraffin diffraction spots at 3.9–4.2 Å resolution at 300 keV and 100 keV. Similarly, Fig. 4 shows the fading of the intensity of the diffraction spots from purple membrane in three resolution zones, namely 20–6.2 Å, 6.2–4.4 Å and 4.4–3.6 Å, and at the two electron energies. In the figure, these zones are labelled 9 Å, 5 Å and 4 Å, representing roughly the centre of each resolution zone.

Apart from a slightly slower rate at the beginning of the exposure series, the purple membrane intensities fade roughly exponentially with fluence, with the plots looking nearly linear on a logarithmic scale. The high-resolution diffraction spots fade more quickly than the low-resolution spots. Fig. 4 shows that the B-factor (since $I=I_{0}e^{-2B/4d^{2}},$ then $B=2d^{2}\ln\left(I_{0}/I\right)$ for spacing *d*) that describes the fall-off reasonably well increases by about 7 Å^2^ for every 1 $e^{-}/$Å^2^ of exposure at 300 keV. For the fading of the paraffin diffraction spots, there are clearly two phases. The intensities appear to fade slowly at first and then more rapidly after 10–20 $e^{-}/$Å^2^ exposure. We believe this might be due to the fact that the amorphous carbon gets wrinkled when it is cooled to liquid nitrogen temperature, so the C_44_H_90_ crystals are not very flat. As a result, we are not sure whether the beam-induced motion and tilting of the C_44_H_90_ crystals as radiation damage builds up might be bringing new regions into the diffracting position (i.e. into intersection with the Ewald sphere), and thus making the diffraction spots appear to fade more slowly in the initial part of the exposure. Negligible movement of the stage occurred during exposure (imaged before and after irradiation). We did not consider if the two stages could be related to structural loss in the paraffin molecules leading to an additional loss of crystallinity between molecules after a certain degree of damage. In later frames, when the crystals have become substantially disordered, and the diffraction spots fuzzier as a result, we think this possibility has receded, giving rise to a more rapid fading in later frames.

In any case, since we are comparing spot fading at the two electron energies in exactly the same way using the same specimen on the same grid in the same microscope with the same detector, we are confident that the comparison of spot fading is a valid measure of radiation damage. To minimise undue bias due to curve fitting or other ways to measure spot fading, we have therefore adopted a simple criterion to measure radiation damage on both specimens. We show in Table 1 the values for *N_e_*, which is defined as the electron fluence (measured in $e^{-}/$Å^2^) at which the spot intensities fade to 1/*e* of their initial value. We estimate *N_e_* from points in the log plots that correspond to 0.5 × , 1.0 ×  or 2.0 ×  ln (1/*e*). From the last column in Table 1, it is clear that radiation damage per incident electron is greater at 100 keV than at 300 keV by a factor of 1.57 ± 0.03.

**Table 1:** Fading of reflection spot intensities from 2D crystals at 80 K. Reported as ratio of diffraction spot fading *N_e_* in $e^{-}$/Å at 100/300 for C_44_H_90_ and purple membrane at different resolutions. Electron fluence *N_e_* for diffraction spot intensities to fall to 1/*e* (the base of natural logarithms) of their initial diffraction intensity is on average 1.57  ±  0.03 times greater at 300 keV than at 100 keV. Measurements of fluence for various multiples *m* of 1/*e* and normalised by *m* in columns 4 and 5 to allowing direct comparison of *N_e_* values with increasing damage.

Specimen	Resolution band (Å)	Multiple of 1/*e*	*N_e_* 100 keV ($e^{-}/$Å^2^)	*N_e_* 300 keV ($e^{-}/$Å^2^)	Ratio *N_e_*(300 keV)/*N_e_*(100 keV)
Paraffin	4	0.5	20.0	29.6	1.48
		1.0	13.3	21.5	1.61
		2.0	9.30	14.5	1.56
Purple	9	0.5	14.8	22.9	1.55
membrane		1.0	12.4	21.0	1.69
		2.0	10.9	–	–
	5	0.5	6.80	9.70	1.43
		1.0	5.72	8.65	1.51
		2.0	5.13	7.73	1.51
	4	0.5	5.50	9.64	1.75
		1.0	4.85	7.77	1.60
		2.0	4.20	7.02	1.67
**Average**					**1.57 ± 0.03**

## 4 Discussion

### 4.1 Damage vs elastic scattering

In this work we have measured the electron elastic and inelastic scattering cross-sections for carbon in the range of 100 to 300 keV to an accuracy of 0.5% and 5% respectively. We have also measured the ratio of the amount of damage per unit electron fluence for crystals of paraffin and purple membrane at 100 keV and 300 keV and find that it matches the inelastic cross-section to within error (1.57  ±  0.03 times higher at 100 keV than 300 keV). Compared to the elastic scattering cross-section ratio (2.01  ±  0.01 times higher at 100 keV than 300 keV), and with the assumption that the electron scattering processes for carbon in this form and in molecules are essentially the same in this regime, this demonstrates that there is about 25% more elastic scattering per unit damage at 100 keV than at 300 keV. The experiments we have described here were specifically designed to try to eliminate other sources of systematic variation between the different energies. Having verified the scaling relationship between the inelastic and elastic cross-sections, and demonstrated that the damage to biological specimens correlates directly with the inelastic cross-section, we can now consider the question of what is the optimal energy for cryoEM.

### 4.2 Contrast vs scattering and specimen thickness

**Fig. 5:**
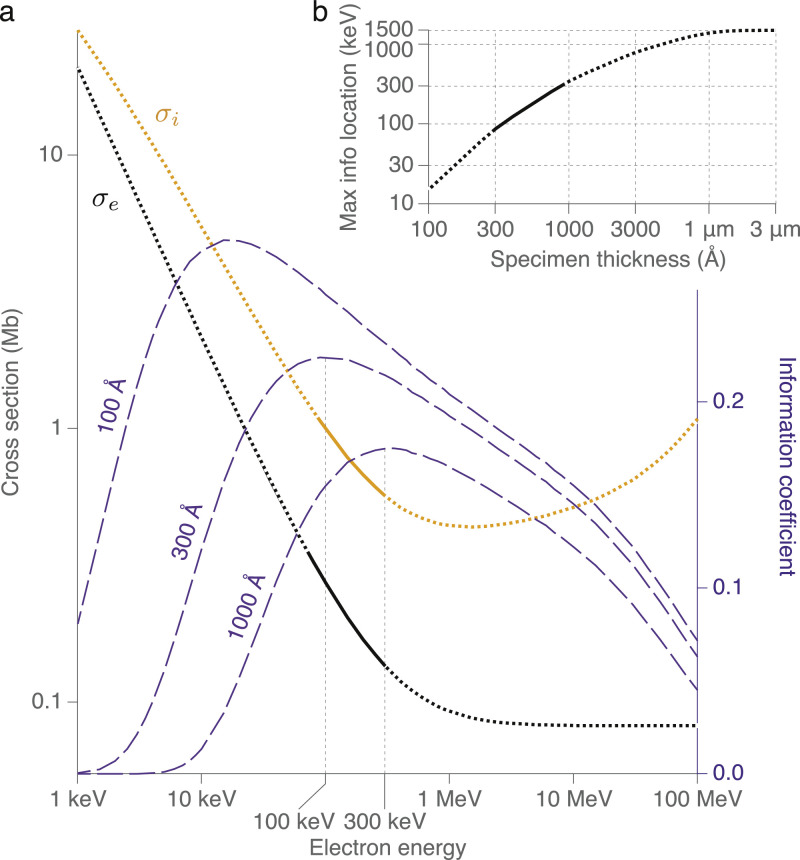
Scaling of cross-sections and information vs energy. The theoretical relationship between the elastic (*σ_e_*) and inelastic (*σ_i_*) scattering cross-sections for carbon are plotted vs. energy. The regions experimentally verified in this work are highlighted with solid lines, with extrapolations in dotted lines. Purple curves are the information coefficient plotted vs energy on the right axis for a 100, 300 and 1000 Å thick specimen. Inset is a plot of the positions of the maxima of the information coefficient vs. thickness, again with the experimental region in solid line. The curve levels off past the minimum in *σ_i_* (minimum ionising particle) as the inelastic cross-section increases at high energy.

Until now, we have only considered atomic cross-sections corresponding to the case where the electron has suffered only one scattering event in transiting the specimen. Graphene is a near perfect test specimen in this respect as it is only one atom thick. Real cryoEM specimens are always thicker than one atom and the effect of this must be considered in determining the best energy for imaging. To do so, we first consider the ratio of the elastic to inelastic scattering cross-sections, as experimentally verified in this work (Fig. 5a). The elastic scattering cross-section follows the theoretical relationship according to [11]

(1) $$\sigma_{e}=\frac{2.1\times 10^{5}}{\beta^{2}}\left[1-\frac{1.6}{137\beta }\right]\;\mathrm{barn}$$

which is plotted in Fig. 5a. Note the second term is a minor correction introduced in Langmore & Smith to improve agreement with measurements particularly at low energy but is less than two percent for the range of energies measured here. Values for the theoretical inelastic scattering cross-section, also plotted in Fig. 5a, are taken from the Estar database [23], which incorporate the best available potential models in a given range of energies.

It is easy to see that the ratio of the elastic scattering to the inelastic scattering (*σ_e_*/*σ_i_*), increases monotonically as the energy is decreased from 1 MeV to 1 keV. So at first blush, lowering the energy to as low as possible gives the best image per unit damage. But in a standard phase contrast TEM, once the electron suffers an inelastic scattering event, it will have lost energy which means it will no longer be focused into the correct image plane due to the chromatic aberration of the objective lens. This in turn means that as the total cross-section increases (or equivalently the specimen increases in thickness) there will be a loss of electrons due to multiple scattering within the specimen. The aberrations of the lenses also have a greater effect on the image at lower energies but we will ignore these for now as they are not the limiting factor at present. To take this thickness effect into account, we define a new quantity which we call the information coefficient

(2) $$\zeta \equiv T\frac{\sigma_{e}}{\sigma_{i}}$$

where *T* is the total transmission through the specimen of thickness *t*, which is approximately equal to

(3) $$T≃e^{-t/\lambda }$$

where *λ* is the total mean free path in the specimen. This quantity is plotted for three thicknesses, 100, 300 and 1000 Å in Fig. 5a. From these plots, one can also find the maximum value of *ζ* for a given specimen thickness, and this is plotted in Fig. 5b.

In the case of cryoEM, the thickness of interest is the thickness of the ice layer containing the specimen; in the optimum, this should be slightly larger than the particle being imaged. Measurements of the effective mean free path of electrons in ice at 300 keV have previously been made. A value of 314 nm was used here, which is in agreement with previous experimental measurements determined by hole drilling technique [24], [25], and a more recent value of 332 nm determined for the inelastically scattered electrons using energy loss spectroscopy [26].

It is interesting to consider the range of thickness over which 100 keV gives a theoretical advantage over the current most commonly used energy (300 keV). Using the calculation shown in Fig. 5, for specimens thinner than about 600 Å, one would be better off imaging at 100 keV from the point of view of radiation damage. This encompasses nearly all single particle specimens which, for molecular masses below 10 MDa, are typically in the range of 50–400 Å in diameter.

In principle, a chromatic aberration (*C_c_*) corrector allows all electrons, even those that have undergone inelastic scattering, to be used for phase contrast imaging since the inelastic electrons are still coherent [27], [28]. Recently constructed *C_c_* corrected TEMs designed for operation in the 20–80 keV [4] and the 80–300 keV [29] ranges demonstrate the feasibility of a *C_c_* corrected electron cryomicrosope. With such an instrument, the maxima of the curves in Fig. 5b would shift to lower energies, such that even relatively thick specimens ( > 3000 Å) could still be effectively imaged using phase contrast with a 300 keV beam. A more detailed calculation of the relative effects of *C_c_, C_s_* and other aberrations, multiple scattering, the magnitude of phase contrast and the application of various approximations including the first Born approximation in the context of the improved theory of radiation damage in cryoEM presented here, would be of great interest but is beyond the present scope. Other considerations like static and dynamic charging of the specimen, and curvature of the Ewald sphere have been recently addressed [30], [31], [32] and are no longer of fundamental concern. Other forms of energy loss begin to become important at high energy (1 MeV and above, see Fig. 5); a direct measurement of the minimum ionisation point predicted for electrons at around 1 MeV would also be of interest to validate the theory in this regime including the asymptotic flattening of the curve in Fig. 5b.

Another important issue to consider in any radiation sensitive context is the electron detector. Current direct electron detectors designed to work well at 300 keV perform poorly at 100 keV, as their performance drops precipitously due to backscattering of incident electrons into pixels distant from where the electron initially enters the detector. This rapidly overwhelms any potential benefit from the increase in the information coefficient described here. Modern phosphor detectors also suffer several loss mechanisms in the cascade of converting the electron to a photon and back again to a charge carrier in the detector chip, which limits their detective quantum efficiency (DQE) at any energy [33]. Further, the needs of a detector for low dose phase contrast imaging are different than for X-ray and STEM/ptychography at similar energies. Work is needed to test direct detector designs with appropriate speed, DQE and pixel size for cryoEM at 100 keV. The development of an inexpensive high-brightness source for use in 100 keV cryomicroscopes is also a priority.

## 5 Conclusions

Based on the measurements presented here, we conclude that with all other considerations being equal, most single-particle cryoEM investigations would benefit from changing the electron energy from 300 to 100 keV instead. The present limitation to low dose imaging at 100 keV is the detector. Currently available direct detectors are either optimised for higher energies (300 keV and above) or lack the combined features required for cryoEM (DQE, number of pixels and frame rate). Widely available electron cryomicroscope optics are sufficient to easily reach  ∼ 2 Å resolution at 100 keV. We therefore expect that for determining the structure of purified biological molecules, the energy of choice will be 100 keV once a suitable detector with high efficiency and speed is widely available.

## Appendix A Supplementary materials

Supplementary Data S1 Supplementary Raw Research Data. This is open data under the CC BY license http://creativecommons.org/licenses/by/4.0/ Supplementary Data S1

Supplementary Data S2 Supplementary Raw Research Data. This is open data under the CC BY license http://creativecommons.org/licenses/by/4.0/ Supplementary Data S2

Supplementary Data S3 Supplementary Raw Research Data. This is open data under the CC BY license http://creativecommons.org/licenses/by/4.0/ Supplementary Data S3

Supplementary Data S4 Supplementary Raw Research Data. This is open data under the CC BY license http://creativecommons.org/licenses/by/4.0/ Supplementary Data S4
